# Epigenetic and transcriptional profiling of triple negative breast cancer

**DOI:** 10.1038/sdata.2019.33

**Published:** 2019-03-05

**Authors:** Andrea A. Perreault, Danielle M. Sprunger, Bryan J. Venters

**Affiliations:** 1 Chemical and Physical Biology Program at Vanderbilt University, Nashville, TN, USA; 2 Department of Molecular Physiology and Biophysics, Vanderbilt Genetics Institute, Vanderbilt Ingram Cancer Center, Vanderbilt University, Nashville, TN, USA

**Keywords:** Histone post-translational modifications, Chromatin immunoprecipitation, Gene expression profiling, Breast cancer

## Abstract

The human HCC1806 cell line is frequently used as a preclinical model for triple negative breast cancer (TNBC). Given that dysregulated epigenetic mechanisms are involved in cancer pathogenesis, emerging therapeutic strategies target chromatin regulators, such as histone deacetylases. A comprehensive understanding of the epigenome and transcription profiling in HCC1806 provides the framework for evaluating efficacy and molecular mechanisms of epigenetic therapies. Thus, to study the interplay of transcription and chromatin in the HCC1806 preclinical model, we performed nascent transcription profiling using Precision Run-On coupled to sequencing (PRO-seq). Additionally, we mapped the genome-wide locations for RNA polymerase II (Pol II), the histone variant H2A.Z, seven histone modifications, and CTCF using ChIP-exo. ChIP-exonuclease (ChIP-exo) is a refined version of ChIP-seq with near base pair precision mapping of protein-DNA interactions. In this Data Descriptor, we present detailed information on experimental design, data generation, quality control analysis, and data validation. We discuss how these data lay the foundation for future analysis to understand the relationship between the nascent transcription and chromatin.

## Background & Summary

Triple negative breast cancer (TNBC) is a highly aggressive and heterogeneous form of cancer^1^. TNBC is characterized by a lack of expression for estrogen receptor (ER), progesterone receptor (PR), and human epidermal growth factor receptor 2 (HER2). Because TNBC lacks these proteins, targeted drug therapies directed against ER, PR, and HER2 are not possible. Therefore, standard care typically includes surgical resection, radiation, and chemotherapy. Prognosis remains poor for TNBC patients receiving standard care, highlighting the need for new and innovative therapeutic strategies. Emerging efforts have focused on the epigenome as a therapeutic target for TNBC^2^. It is now clear that epigenetic mechanisms play an important role in the pathogenesis, maintenance, and therapeutic resistance of the disease^3^. Consistent with this notion, a recently proposed model for transcriptional addiction in cancer suggests that transcriptional and chromatin regulators are potential targets for therapeutic intervention using innovative approaches^4^.

Given that epigenetic and transcriptional regulators are implicated in the pathogenesis of TNBC, there is a need to better understand the mechanisms involved in the epigenetic modulation of genes expressed in TNBC^5^. Functional genomic approaches offer an unbiased, comprehensive glimpse into how transcriptional and chromatin regulators interact with the genome. For example, nascent transcriptional profiling (Precision Run-On sequencing, PRO-seq) and protein-DNA contact profiling (Chromatin Immunoprecipitation Exonuclease and sequencing, ChIP-exo) are state-of-the-art functional genomic tools that enable interrogation of global RNA synthesis and protein-DNA interactions, respectively^6,7^. PRO-seq and ChIP-exo studies generate scientifically valuable datasets due to their unique characteristics and reanalysis potential, which advances the sharing and reuse of scientific data.

PRO-seq and other nascent profiling approaches, such as GRO-seq, are more sensitive at detecting transcriptional responses than traditional assays like RNA-seq because they measure newly synthesized RNA, rather than the steady-state abundance of total RNA synthesis and degradation. This distinction is critical for detecting rapid transcriptional responses to stimuli, such as hormones and drug treatments^8,9^. In addition to using PRO-seq data to compute RNA synthesis rates^10^, other biological features may be inferred from the unique data structure, such as transcription factor (TF) binding and enhancer activity^11–13^. Furthermore, computational tools have been developed to mine PRO-seq (or related GRO-seq) data and provide de novo annotation of long noncoding RNAs, microRNAs, and enhancer RNAs^14–17^. Taken together, the reanalysis potential of PRO-seq data is high.

ChIP-exo displays improved resolution and sensitivity over the traditional ChIP-seq method^18^. Rather than sequencing from the distal sonication borders as in ChIP-seq, ChIP-exo enriched DNA fragments are sequenced from the left and right 5’ DNA borders of the protein-DNA crosslink site. The precision of the resulting data can be leveraged to provide unique and ultra-high resolution insights into the functional organization of the genome^19^. For example, ChIP-exo was uniquely capable of spatially resolving divergent, initiating, paused, and elongating RNA polymerase II (Pol II) on a genome-wide scale^20–22^.

**Figure 1:**
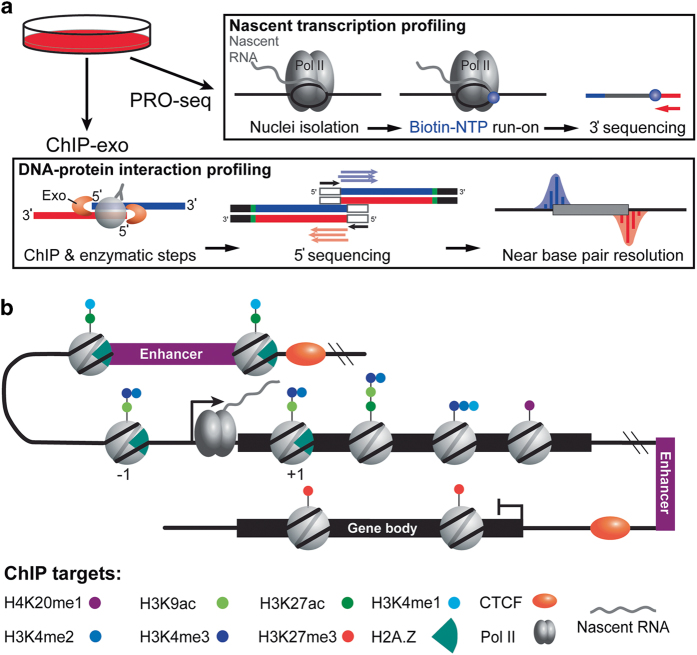
Experimental design and overview of ChIP targets. (**a**) HCC1806 cells were cultured and harvested for PRO-seq and ChIP-exo. PRO-seq measures nascent transcription. ChIP-exo identifies the exonuclease left and right borders that flank protein-DNA interactions. (**b**) Illustration of the genomic context for ChIP targets: Pol II, H2A.Z, H3K4me3, H3K4me2, H3K4me1, H3K27ac, H3K9ac, H3K27me3, H4K20me1, and CTCF.

**Figure 2:**
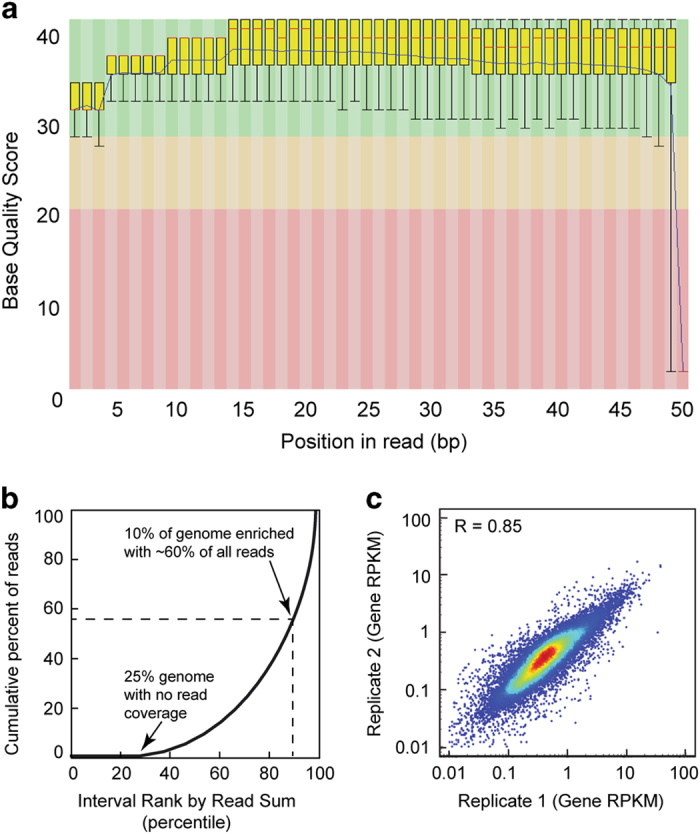
Quality control, enrichment analysis, and reproducibility for PRO-seq and ChIP-exo data. (**a**) Box-plot distribution of base quality scores are shown for H2A.Z ChIP-exo replicate 2. A score greater than 30 (green region) indicates a high confidence base call. (**b**) ChIP enrichment analysis plot that displays the cumulative percent of total reads found in a given percent of the mappable human genome. No ChIP enrichment would result in a diagonal trace. (**c**) Scatter plot correlation analysis for H2A.Z ChIP-exo biological replicates as measured by the Spearman correlation coefficient R-values (upper left corner).

**Figure 3:**
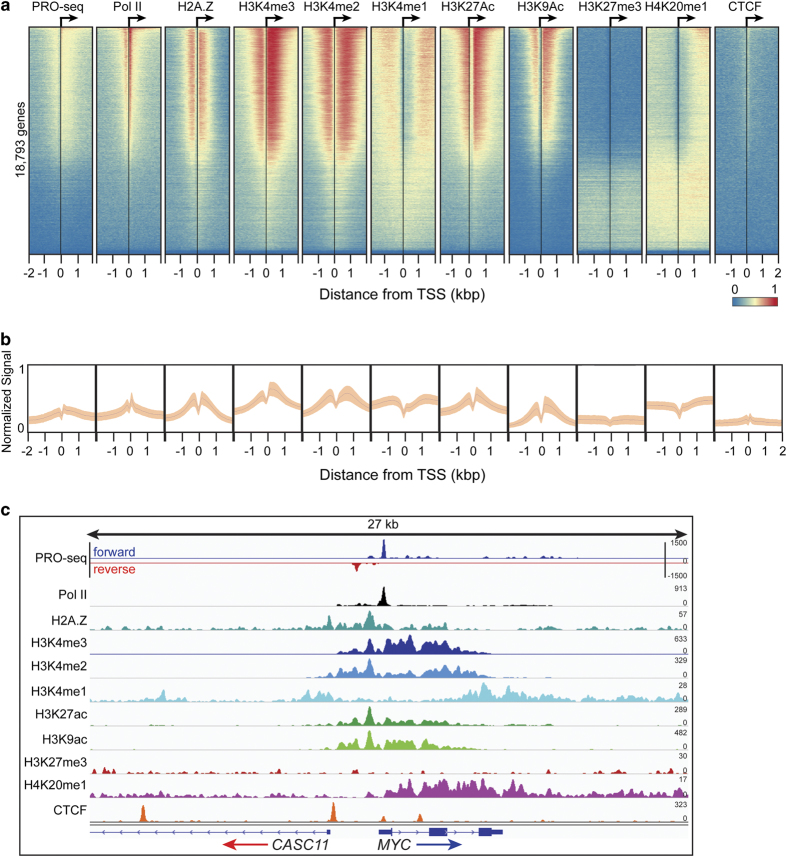
Genomic distribution of RPKM normalized signal for PRO-seq and ChIP-exo targets (Pol II, H2A.Z, H3K4me3, H3K4me2, H3K4me1, H3K27ac, H3K9ac, H3K27me3, H4K20me1, and CTCF). (**a**) Row-linked heatmaps show RPKM normalized number of reads across a 4 kb genomic interval in 40 bp bins relative to the TSS. Heatmaps were generated from merged biological replicate pairs for each data set. Regions are sorted in descending order based on average row tag density for Pol II. Each row represents a gene, with 18,793 genes displayed. Red and blue reflect high and low read densities, respectively. (**b**) Composite plots below each heatmap quantify the normalized tag density. The central trace denotes the average tag density for each 40 bp bin and the orange fill reflects the standard deviation. (**c**) Genome browser view of PRO-seq and ChIP-exo signal for the indicated targets in HCC1806 cells shown at the *MYC* gene. Tag distributions were smoothed and RPKM normalized using deepTOOLS. Traces were generated from merged biological replicate pairs.

In this Data Descriptor, we provide a technical validation of twenty-two functional genomic data sets that interrogate the nascent transcription, Pol II binding, and chromatin architecture using the HCC1806 preclinical model for TNBC^24–26^. In this study, we focused on the TNBC HCC1806 cell line and have generated 2 PRO-seq data sets and 20 ChIP-exo data sets (2 biological replicates for each of the following targets: Pol II, H2A.Z, H3K4me3, H3K4me2, H3K4me1, H3K27ac, H3K9ac, H3K27me3, H4K20me1, and CTCF). ChIP-exo mapping of Pol II, a histone variant, and select histone modifications should enable other investigators to use these data sets for their own research to further understand the detailed interplay of Pol II and chromatin in ultra-high resolution in a preclinical model for breast cancer. On average, 36 million uniquely aligned reads were generated for each PRO-seq and ChIP-exo data set (Table 1). To facilitate interpretation of these data, we provide detailed information on experimental design (Fig. 1), sequence quality control analyses (Fig. 2), and biological validation (Fig. 3).

## Methods

### Tissue culture

The human HCC1806 triple negative breast cancer cell line, basal-like 2 subtype (ATCC) was maintained at 37 ^°^C in 5% CO_2_ between 20–80% confluency in RPMI 1640 (Roswell Park Memorial Institute, Gibco 11875-093) containing 10% bovine calf serum (Gibco 16170-078), 1% L-glutamine (Gibco 25030-081), and 1% Penicillin/Streptomycin (Gibco-15146-122).

### PRO-seq library preparation

PRO-seq was performed as previously described^7^ with isolated nuclei from 25 million cells from two biological replicates. To enable comparisons to drug treatment experiments, cells were treated with vehicle (final 0.03% DMSO (dimethyl sulfoxide)) for 4 h prior to harvest.

### ChIP-exo library preparation

ChIP-exo was performed as previously described^6,20^ with chromatin extracted from 50 million cells, ProteinG MagSepharose resin (GE Healthcare), and 5 ug of antibody directed against RNA polymerase II (Santa Cruz sc899), H2A.Z (EMD Millipore 07-594), H3K4me3 (Abcam ab8580), H3K4me2 (Abcam ab7766), H3K4me1 (Abcam ab8895), H3K27ac (Abcam ab4729), H4K9Ac (Abcam ab4441), H3K27me3 (EMD Millipore 07-449), H4K20me1 (Abcam ab9051), and CTCF (EMD Millipore 07-729). Two biological replicates were prepared for each ChIP target. To enable future comparisons to drug treatment experiments, cells were treated with vehicle (final 0.03% DMSO (dimethyl sulfoxide)) for 4 h prior to harvest. Libraries were sequenced using an Illumina NextSeq500 sequencer as single-end reads 50 or 75 nucleotides in length (Table 1).

### Sequence read alignment and quality control

The base call quality for each sequenced read was assessed using the FastQC program (bioinformatics.babraham.ac.uk/projects/fastqc/) (Fig. 2a and Supp. Figs. 1–3). Sequence reads (fastq files) were aligned to the human hg19 reference genome build using BWA-MEM algorithm with default parameters^27^. The resulting bam files were first sorted using the Samtools Sort function, and then bam index files were generated using the Samtools Index function^28^. The purpose of bam index files is to enable viewing of raw sequencing data in a genome browser. Next, genome-wide read coverage and enrichment were assesses using deepTOOLS fingerprint plots^29^ (Fig. 2b and Supp. Figs 4–6).

### Biological validation

To estimate variance across biological replicates, the Pearson correlation coefficient for pairwise gene Reads Per Kilobase of genome per Million reads (RPKM) was computed (Fig. 2c, Supp. Fig. 7) using the HOMER (Hypergeometric Optimization of Motif EnRichment) suite^30^. Briefly, bam files were converted to tag directories using the makeTagDirectory function with the –genome, –checkGC, and –format options. To quantify and normalize tags within gene body regions to RPKM, the analyzeRepeats function was used with the –rpkm and –d options (2019SciDataVenters_RPKM.xlsx, Data Citation 1).

bedTOOLS was used to convert files from bam to Bigwig, and then the ChAsE (Chromatin Analysis and Exploration) suite was used to display the read distribution relative to the TSS from Bigwig files (Fig. 3a,b, Supp. Fig. 8)^33^. Raw sequencing tags were binned, smoothed, and RPKM computed using the deepTOOLS genomeCoverage tool (20 bp bin, 100 bp sliding window)^29^. Smoothed RPKM signal was visualized with Integrative Genomics Viewer (IGV) (Fig. 3c)^32^.

### Code availability

Below is a list of software used in this study.

FastQC v0.11.2 (www.bioinformatics.babraham.ac.uk/projects/fastqc/)

^27^BWA-MEM v0.7.13

^28^Samtools v1.3.1

^30^HOMER v4.6

^31^ChAsE v1.0.11

^29^deepTOOLS v2.2.4

^33^bedTOOLS v2.24.0

^32^IGV v2.3.77.

## Data Records

PRO-seq and ChIP-exo bigwig data files from merged replicates were deposited in the NCBI Gene Expression Omnibus (GEO) (Data Citation 2). GEO linked PRO-seq and ChIP-exo bam data files for each replicate were deposited in the Sequence Read Archive (SRA) (Data Citation 3). Table 1 contains sequencing statistics for each data set and linked to its SRA identification number.

## Technical Validation

### Overview of experimental design

In this study, functional genomic experiments using HCC1806 cells were designed with two primary goals in mind. First, PRO-seq data sets were generated to specifically measure nascent transcription. Second, the ChIP targets (Pol II, H2A.Z, H3K4me3, H3K4me2, H3K4me1, H3K27ac, H3K9ac, H3K27me3, H4K20me1, and CTCF) were selected so that Pol II binding and chromatin architecture may be examined on a genome-scale at high precision (Fig. 1). Histone modifications follow patterns of enrichment and delineate specific regions in the genome. For example, H3K4me1 and H3K27Ac are known to be found around distal enhancer regions, along with the histone variant H2A.Z. H3K4me1/2/3 and H3K9Ac are associated with promoter proximal regions, where H3K27ac and H2A.Z are also present^23,34–43^. H4K20me1 is critical for proper cell cycle progression and is typically depleted at promoters, but enriched in the body of genes^44^. Repressive marks and structural transcription factors, such as H3K27me3 and CTCF respectively, insulate regions of the genome that are not actively transcribed^35,45,46^. Taken together, reanalysis of this collection of data should enable new biological insights into chromatin dynamics in a pre-clinical breast cancer model. Below, we briefly describe the rationale and considerations for sequencing data analysis with respect to general read quality, genome alignment, ChIP enrichment, replicate correlation, and biological validation.

### Raw sequence quality control analyses

To assess the quality of the raw sequencing data sets, base call scores were analyzed using the FastQC program and displayed as a box plot distribution at each base position (Fig. 2a and Supp. Figs. 1–3). The average base quality score for a majority of the data sets in the present study fell within the high confidence range (base quality score of 30–40, green region).

Raw sequence reads were aligned to the hg19 build of the human genome. On average, 46 million total aligned reads were generated for each PRO-seq and ChIP-exo data sets (Table 1). Because of the ambiguity of reads that align to multiple locations throughout the genome, we only retain uniquely aligned reads for subsequent analyses. On average, 36 million uniquely aligned reads were obtained per data set, representing an average unique alignment rate of 76%.

Two critical questions for assessing ChIP sequencing data quality are: 1) how much of the genome is represented by a given experiment? and 2) to what extent did the ChIP assay enrich for specific regions of the genome? Typically, high genome coverage and strong ChIP enrichment are desirable in ChIP experiments. To determine genome coverage and ChIP enrichment simultaneously, we used the deepTOOLS suite to perform a fingerprint analysis (Fig. 2b). In the case of H2A.Z ChIP-exo (Fig. 2b), the fingerprint plot trace intersects the x-axis at 25, indicating 75% genome coverage. In fingerprint plots, a rightward deflection of the trace indicates the extent of ChIP enrichment. Given a point along the trace that is the point of intersection from the axes, the corresponding values on the x- and y-axes denote the percent of genome and the percent of all uniquely aligned reads, respectively. Together, these values reflect ChIP enrichment.

For example, the H2A.Z ChIP-exo fingerprint trace reveals that 10% of the genome is enriched with 60% of all uniquely aligned reads, suggesting strong enrichment in the H2A.Z ChIP-exo data set (Fig. 2b). Fingerprint plots for other replicates showed similar patterns of genome coverage and ChIP enrichment (Supp. Figs 4–6). Theoretically, complete genome coverage with no enrichment would be result in a trace with a slope equal to one that intersects the origin (eg: whole genome sequencing wherein 50% of the genome is contains 50% of all aligned reads).

### Biological validation

After verifying the quality of the raw sequencing data, we next sought to provide evidence of biological validity for the data. First, we determined the extent to which biological replicates were reproducible using correlation scatter plots (Fig. 2c). For each gene, the RPKM was computed using the HOMER suite (Data Citation 1). Pearson correlation coefficients (R values) were computed for pairwise correlation plots of gene RPKM across biological replicates. For example, biological replicates for H2A.Z ChIP-exo analysis displayed an R value of 0.85, indicating high reproducibility (Fig. 2c). Overall, correlation analysis resulted in an average R-values of 0.86 (Supp. Fig. 7). Because CTCF is typically enriched in intergenic regions rather than within gene bodies, correlation analysis compared peak RPKM.

Given that certain histone modifications are consistently found at distinct regions of the stereotypical gene, analyzing global patterns of ChIP signal relative to TSSs is a useful method to assess biological validation^47^. For example, it is well established that once Pol II initiates transcription of genes in metazoans, Pol II moves into a stable paused state 30–50 bp downstream of the TSS. Nascent transcription profiling with PRO-seq enables quantification of RNA synthesis and largely coincides with Pol II ChIP-exo density. H3K4me1/2/3 and H3K9Ac are associated with promoter proximal regions, surrounding the TSS of active genes in combination with H3K27Ac. The histone variant H2A.Z is consistently incorporated into the + 1 nucleosome of actively transcribed genes. Distal to gene promoters, H3K4me1 and H3K27Ac have been used as predictive marks of enhancers, which regulate the transcription of their target genes in a distance and orientation independent manner.

Thus, to examine global patterns of ChIP enrichment, the Chromatin Analysis and Exploration (ChAsE) heatmap tool was used to align ChIP signal merged from both biological replicates to TSSs (Fig. 3a, sorted by max peak; and Supp. Fig. 8, sorted by max peak position). Quantification of signal density relative to TSSs is displayed as a composite plot below each heatmap (Fig. 3b). As expected, Pol II was strongly enriched at the pause site just downstream of the TSS. H2A.Z enrichment at the -1 and + 1 nucleosomes immediately flanked Pol II density. H3K4me2, H3K4me3, and H3K9ac were enriched at the + 1 nucleosome as well, but also spread into the body of gene, overlapping the + 1, + 2, and + 3 nucleosome positions. Interestingly, H3K27ac density was similar to H3K9ac but avoided the + 1 nucleosome position. H3K4me1 and H4K20me1 density excluded promoter regions, but were enriched further downstream into the gene body. In contrast to the other histone modifications and consistent with its association to gene repression, H3K27me3 was enriched at genes with the least Pol II and PRO-seq density. Lastly, as expected, gene bodies largely lacked CTCF signal. To examine individual examples of global patterns, RPKM normalized tracks for PRO-seq and ChIP-exo signal were displayed using the Integrative Genome Viewer (IGV), and displayed at the *MYC* gene locus (Fig. 3c). Finally, a comparison of Pol II ChIP-exo and ChIP-seq signal at promoters (Supp. Fig. 9) shows that Pol II ChIP-exo more clearly resolves adjacent peaks of Pol II enrichment corresponding to Pol II pausing just after the TSS and divergent transcription just upstream of the TSS. This is evident both as a genome-wide pattern (Supp. Fig. 9a,b) and in several anecdotal examples (Supp. Fig. 9c).

Taken together, the data presented in this Data Descriptor represents high quality next generation sequencing data that are biologically valid, and should be useful to future studies that seek to understand the interplay of Pol II and chromatin in high resolution on a global scale.

## Additional information

**How to cite this article**: Perreault, A. A. *et al*. Epigenetic and transcriptional profiling of triple negative breast cancer. *Sci. Data*. 6:190033 https://doi.org/10.1038/sdata.2019.33 (2019).

**Publisher’s note**: Springer Nature remains neutral with regard to jurisdictional claims in published maps and institutional affiliations.

## Supplementary Material



Supplementary Information

## Figures and Tables

**Table 1:** Sequencing read alignment statistics for PRO-seq and ChIP-exo data sets.

ChIP target	Antibody	Replicate	SRA identification	Read Length	Total Mapped Reads	Uniquely Mapped Reads	Unique Mapping Rate
**PRO-seq**		1	SRX4485383	50	46,406,885	35,062,933	76%
		2	SRX4485400	75	87,474,762	43,763,320	50%
		**TOTAL**			**133,881,647**	**78,826,253**	
**Pol2**	sc899 (Santa Cruz)	1	SRX4485384	50	82,965,774	63,381,472	76%
		2	SRX4485397	50	32,051,539	24,838,833	77%
		**TOTAL**			**115,017,313**	**88,220,305**	
**H2A.Z**	07-594 (EMD Milipore)	1	SRX4485385	50	9,783,691	6,506,998	67%
		2	SRX4485398	50	41,880,546	31,974,125	76%
		**TOTAL**			**51,664,237**	**38,481,123**	
**H3K4me3**	ab8580 (Abcam)	1	SRX4485387	50	37,105,094	30,487,398	82%
		2	SRX4485404	75	76,560,088	59,872,974	78%
		**TOTAL**			**113,665,182**	**90,360,372**	
**H3K4me2**	ab7766 (Abcam)	1	SRX4485388	50	28,077,305	21,915,585	78%
		2	SRX4485401	75	74,333,953	58,377,458	79%
		**TOTAL**			**102,411,258**	**80,293,043**	
**H3K4me1**	ab8895 (Abcam)	1	SRX4485389	50	43,663,792	32,717,084	75%
		2	SRX4485402	50	37,395,250	28,813,014	77%
		**TOTAL**			**81,059,042**	**61,530,098**	
**H3K27Ac**	ab4729 (Abcam)	1	SRX4485390	50	32,203,255	17,535,368	54%
		2	SRX4485395	50	43,865,531	31,734,234	72%
		**TOTAL**			**76,068,786**	**49,269,602**	
**H3K9Ac**	ab4441 (Abcam)	1	SRX4485392	50	30,684,340	26,874,881	88%
		2	SRX4485394	50	16,339,334	14,037,513	86%
		**TOTAL**			**47,023,674**	**40,912,394**	
**H3K27me3**	07-449 (EMD Millipore)	1	SRX4485391	50	36,950,654	29,977,079	81%
		2	SRX4485396	75	37,579,106	32,496,728	86%
		**TOTAL**			**74,529,760**	**62,473,807**	
**H4K20me1**	ab9051 (Abcam)	1	SRX4485399	75	79,587,575	62,260,072	78%
		2	SRX4485393	75	44,993,047	36,175,837	80%
		**TOTAL**			**124,580,622**	**98,435,909**	
**CTCF**	07-729 (EMD Millipore)	1	SRX4485386	75	65,862,116	48,218,698	73%
		2	SRX4485403	75	61,974,400	52,856,699	85%
		**TOTAL**			**127,836,516**	**101,075,397**	
