# Supplementary material for: Epigenetic and transcriptional profiling of triple negative breast cancer
Source: Sci Data. 2019 Mar 5;6:190033. doi: 10.1038/sdata.2019.33 (PMC6400101; doi:10.1038/sdata.2019.33)
Supplement: Supplementary Information [file sdata201933-s2.pdf]

Supplementary Figures for:

Andrea A. Perreault, Danielle M. Sprunger, and Bryan J. Venters. Epigenetic and transcriptional profiling of triple negative breast cancer. *Nature Scientific Data*. (2019)

Table of Contents

**Figure title**	**Page number**
1. FastQC base quality analysis for biological replicates of PRO-seq and ChIP-exo data sets	2
2. Continued FastQC base quality analysis for biological replicates of ChIP-exo data sets	3
3. Continued FastQC base quality analysis for biological replicates of ChIP-exo data sets	4
4. Fingerprint plots to assess genome coverage and enrichment for PRO-seq and ChIP-exo data sets	5
5. Continued fingerprint plots to assess genome coverage and enrichment for ChIP-exo data sets	6
6. Continued fingerprint plots to assess genome coverage and enrichment for ChIP-exo data sets	7
7. Scatter plot correlation analysis for ChIP-exo biological replicates as measured by the Spearman correlation coefficient R-values (upper left corner)	8
8. ChAsE heatmap display for PRO-seq and ChIP-exo signal from merged biological replicates. Rows are linked and sorted by Pol II ChIP-exo max peak position	9
9. Comparison of Pol II ChIP-exo and ChIP-seq resolution at promoters.	10

Supplementary Figures and Legends

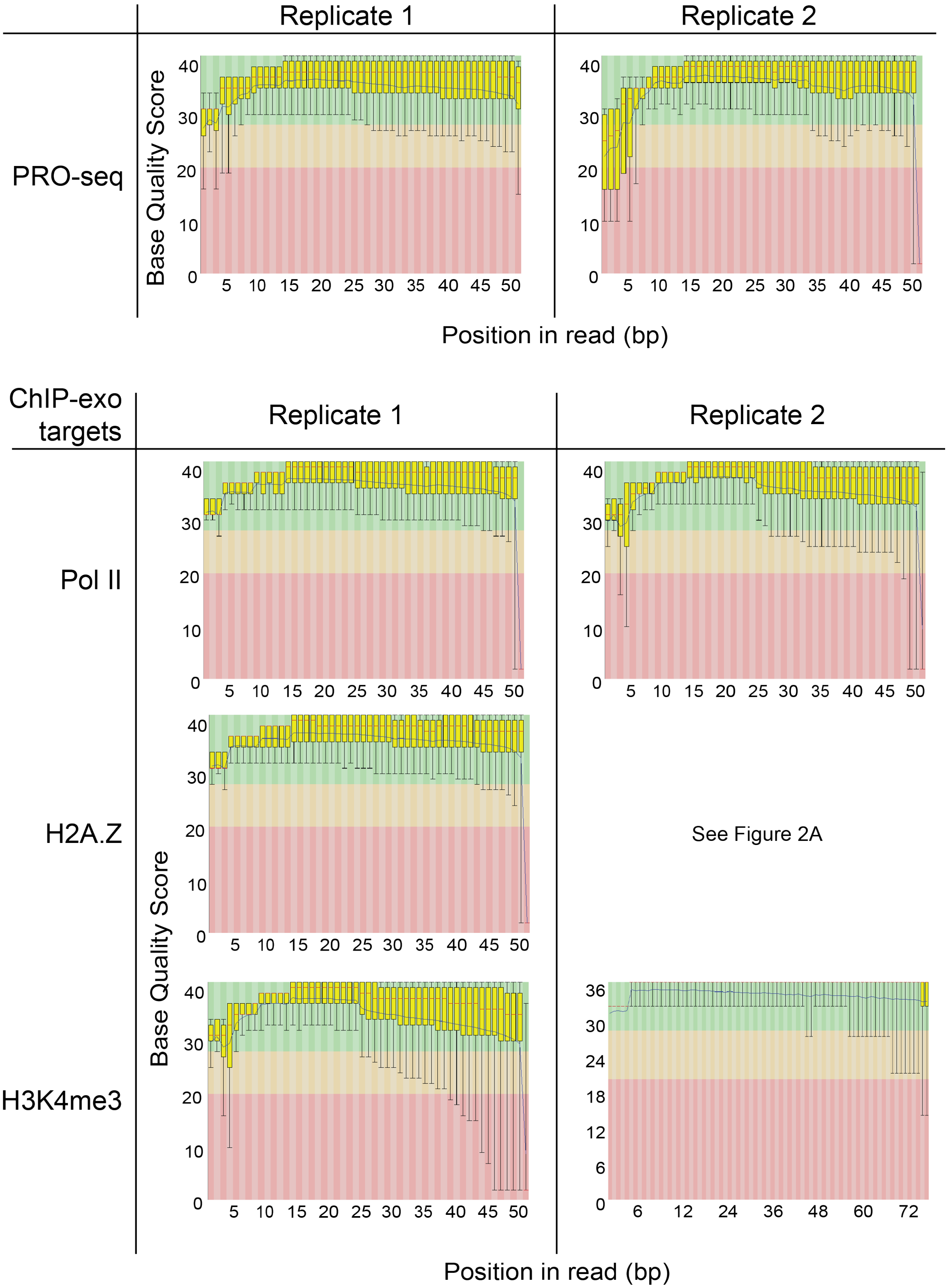

Supp. Fig. 1. FastQC base quality analysis for biological replicates of PRO-seq and ChIP-exo data sets. The mean base quality score is displayed as a blue trace. Related to Fig. 2a.

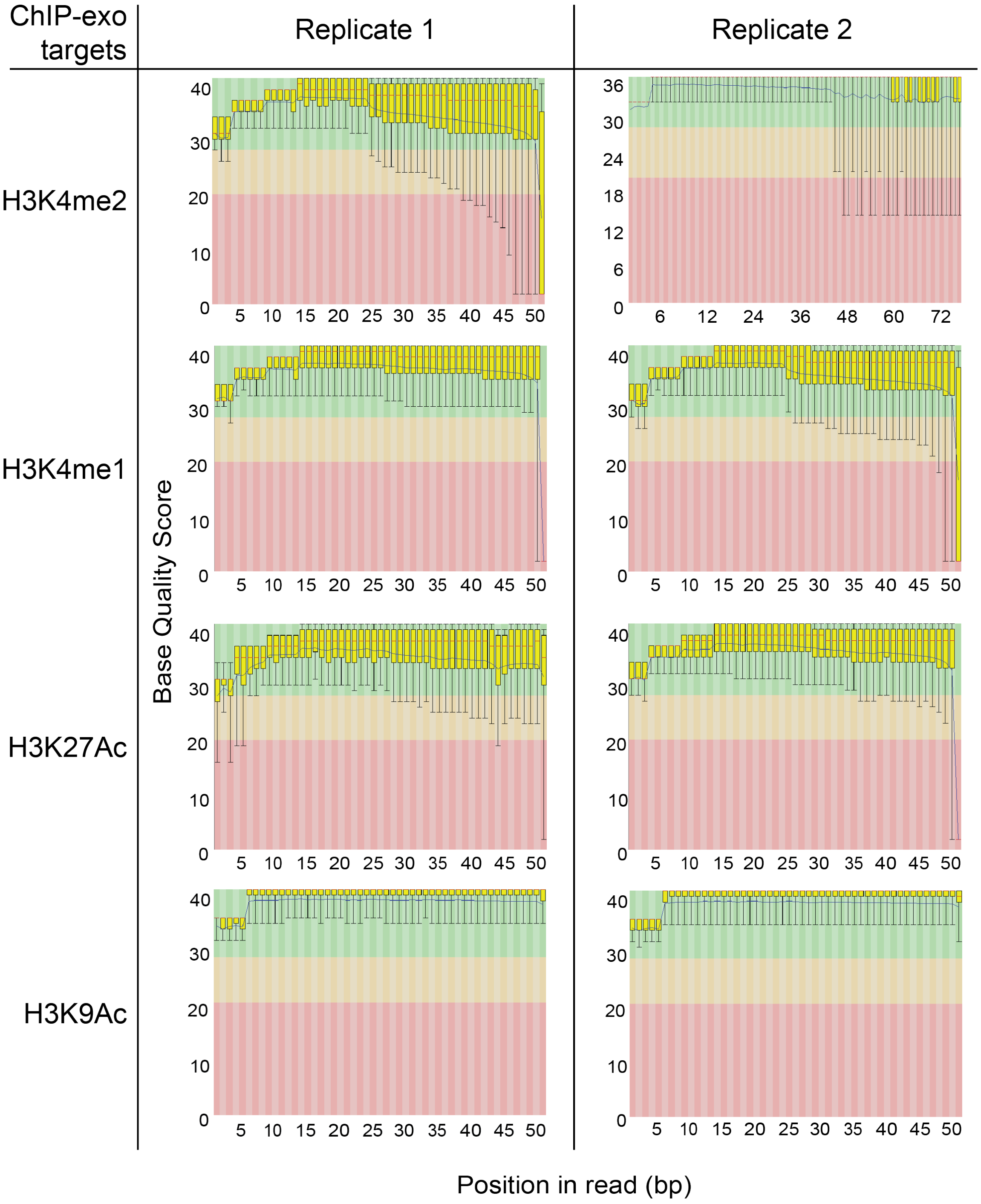

Supp. Fig. 2 Continued FastQC base quality analysis for biological replicates of ChIP-exo data sets. The mean base quality score is displayed as a blue trace. Related to Fig. 2a.

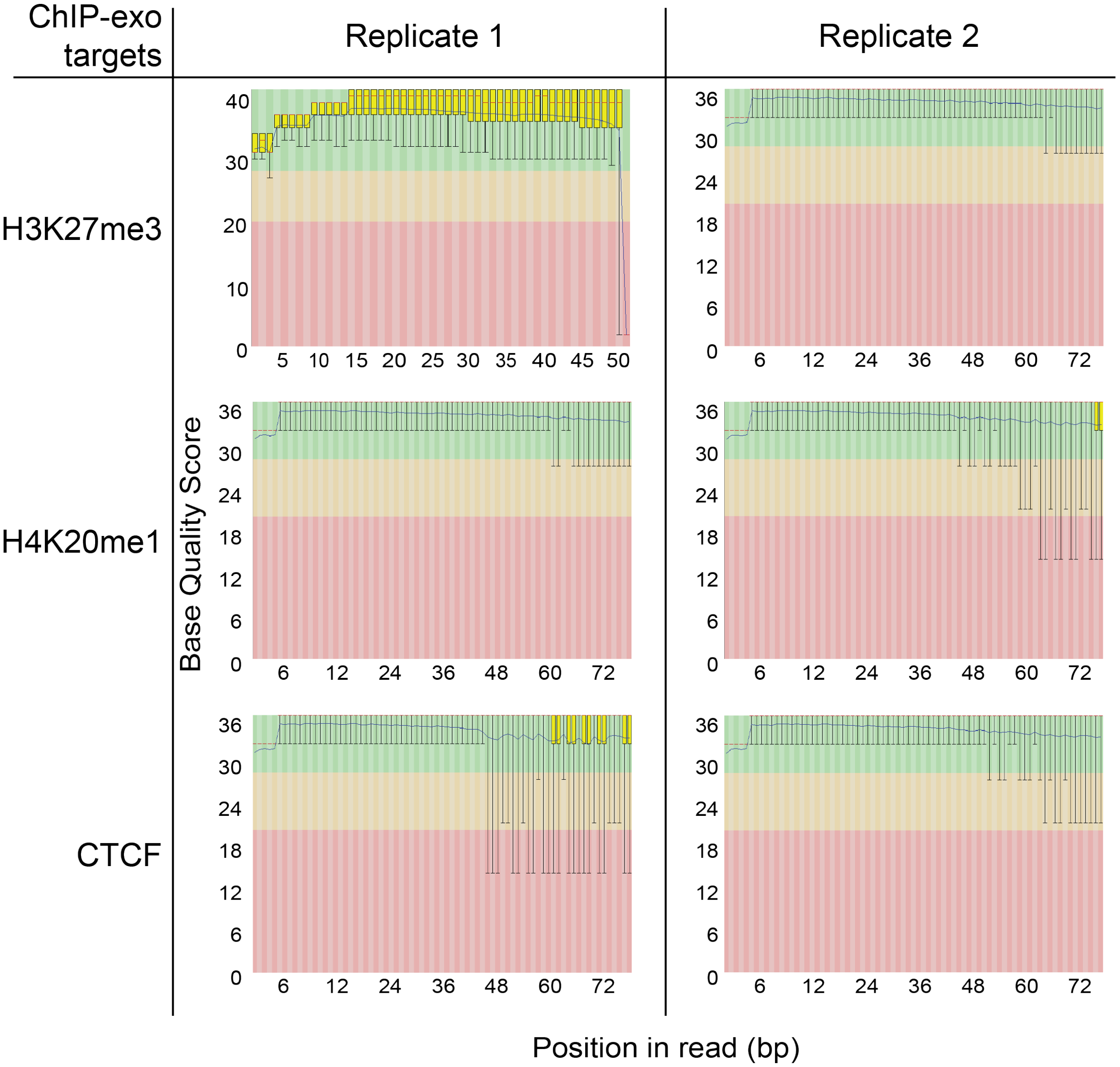

Supp. Fig. 3. Continued FastQC base quality analysis for biological replicates of ChIP-exo data sets. The mean base quality score is displayed as a blue trace. Please note that “box” portion of the box plot is absent at many base positions because the underlying base scores display the same value for their median and quartile ranges. Related to Fig. 2a.

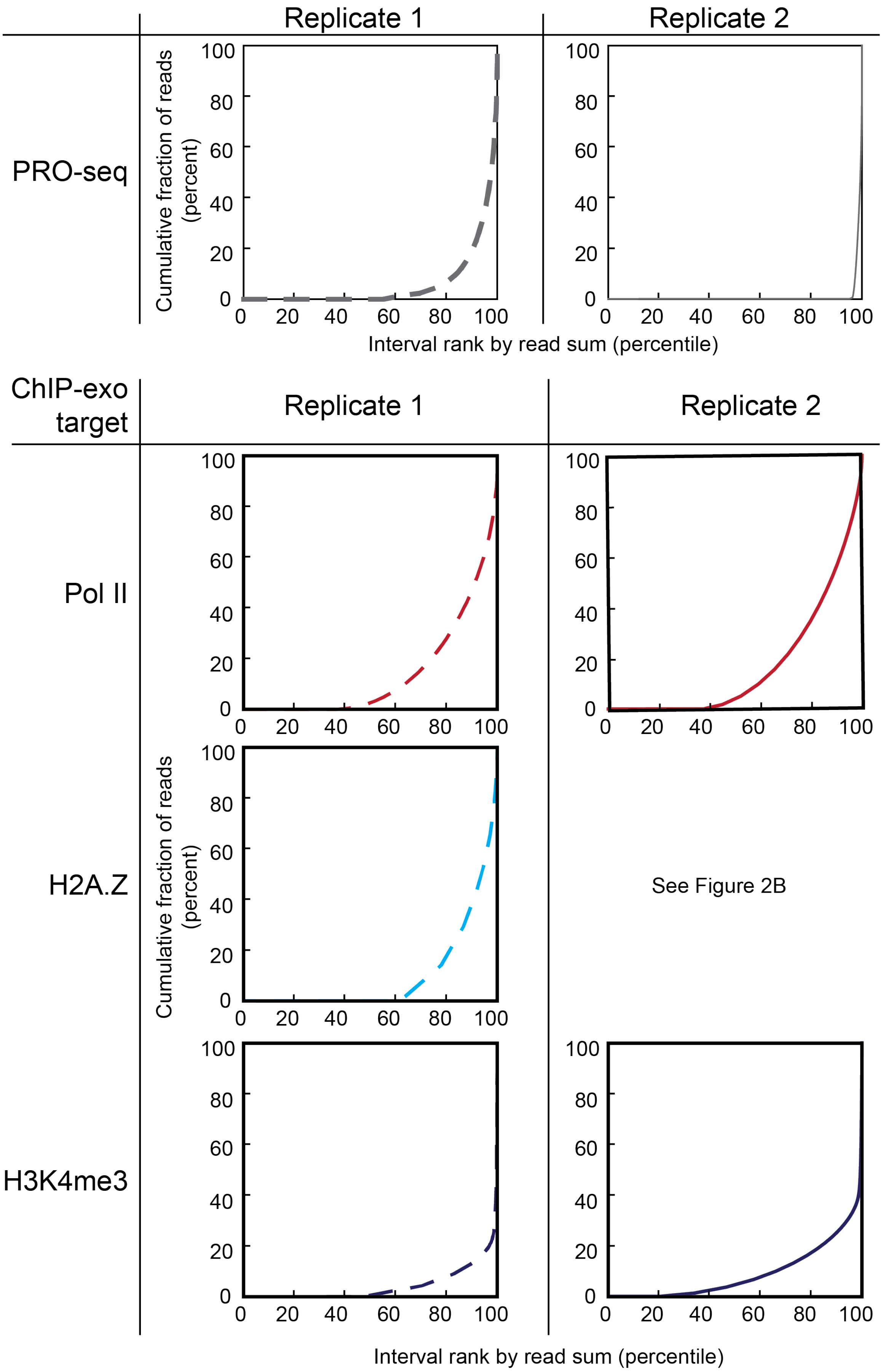

Supp. Fig. 4. Fingerprint plots to assess genome coverage and enrichment for PRO-seq and ChIP-exo data sets. Related to Fig. 2b.

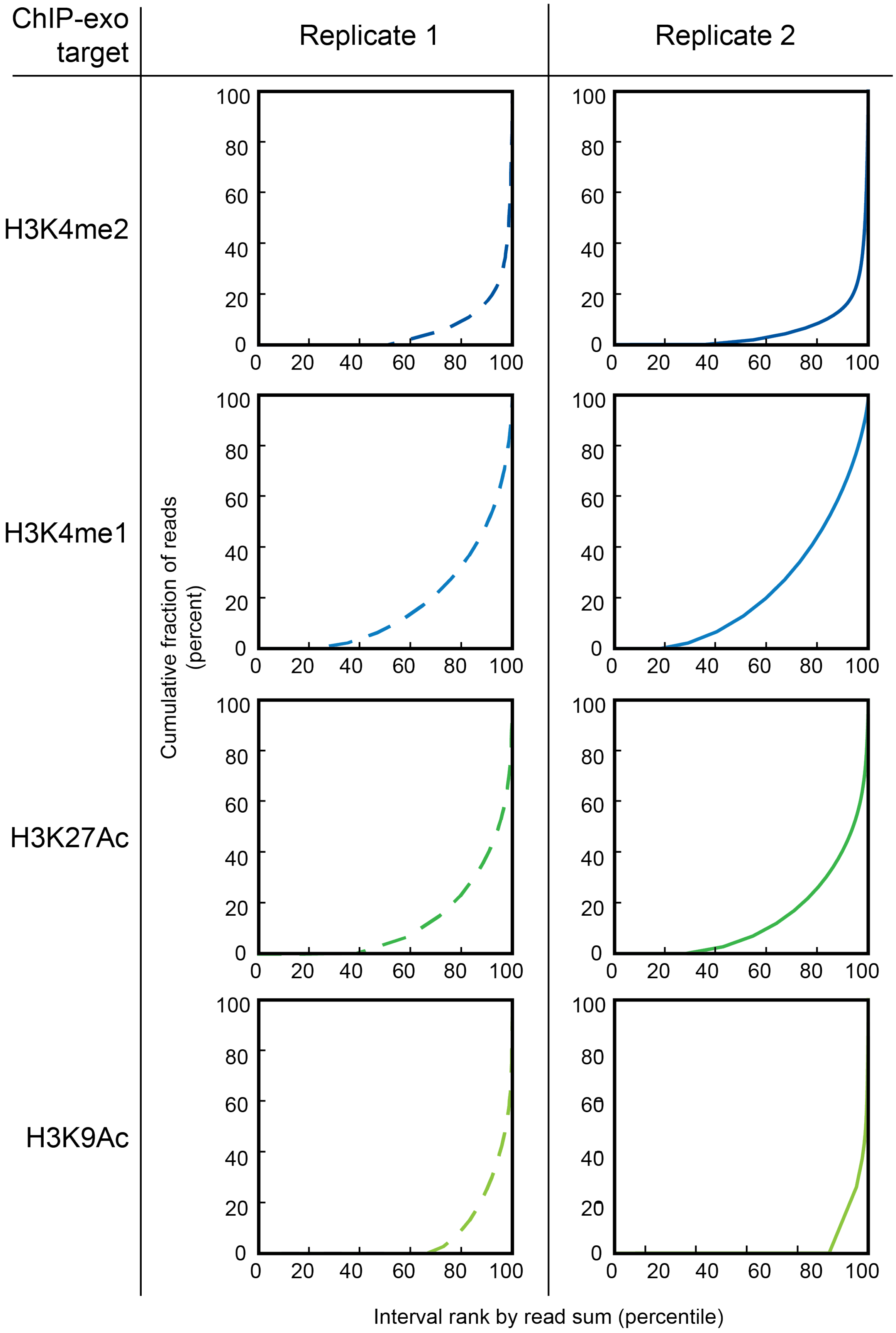

Supp. Fig. 5. Continued fingerprint plots to assess genome coverage and enrichment for ChIP-exo data sets. Related to Fig. 2b.

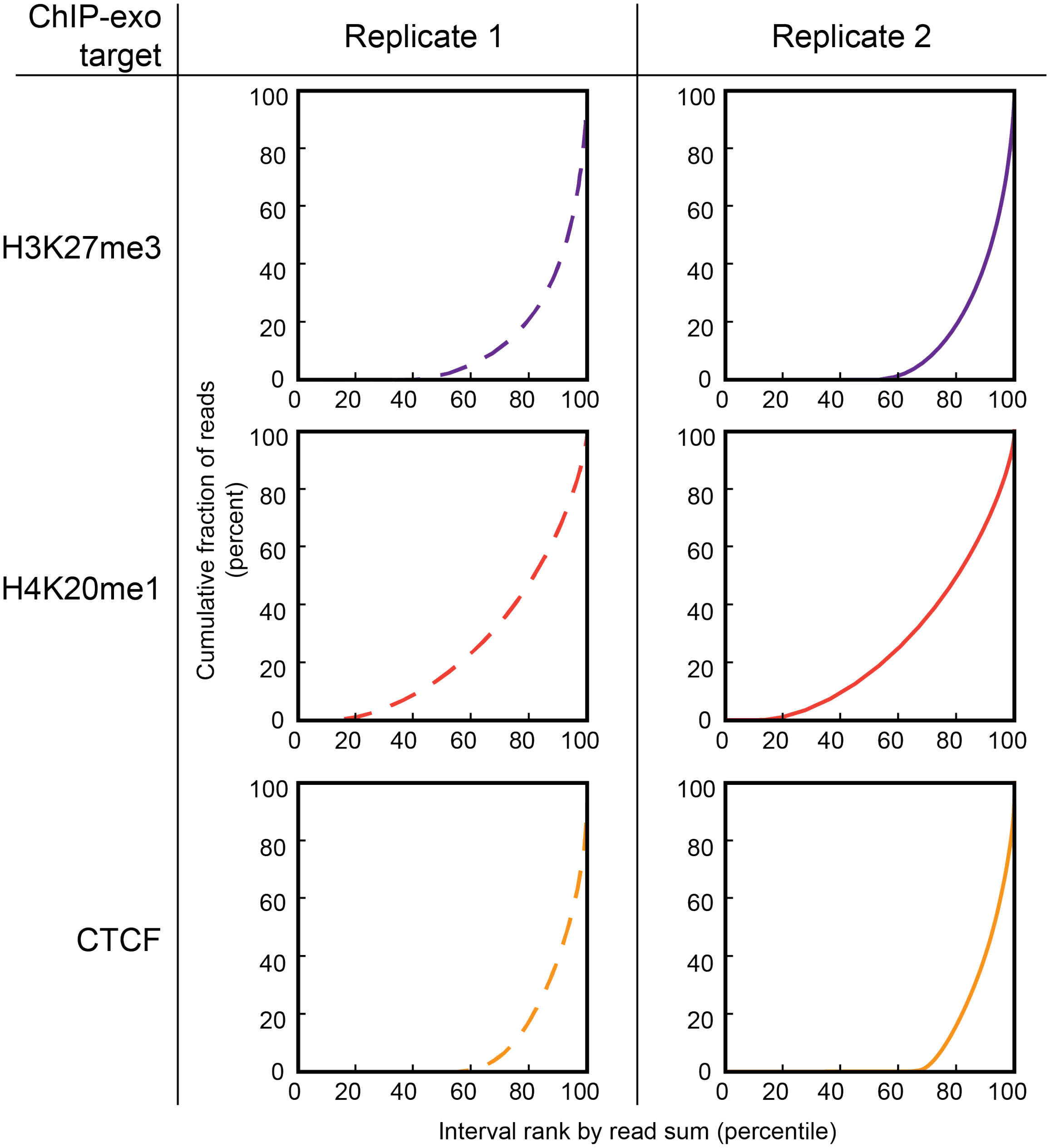

Supp. Fig. 6. Continued fingerprint plots to assess genome coverage and enrichment for ChIP-exo data sets. Related to Fig. 2b.

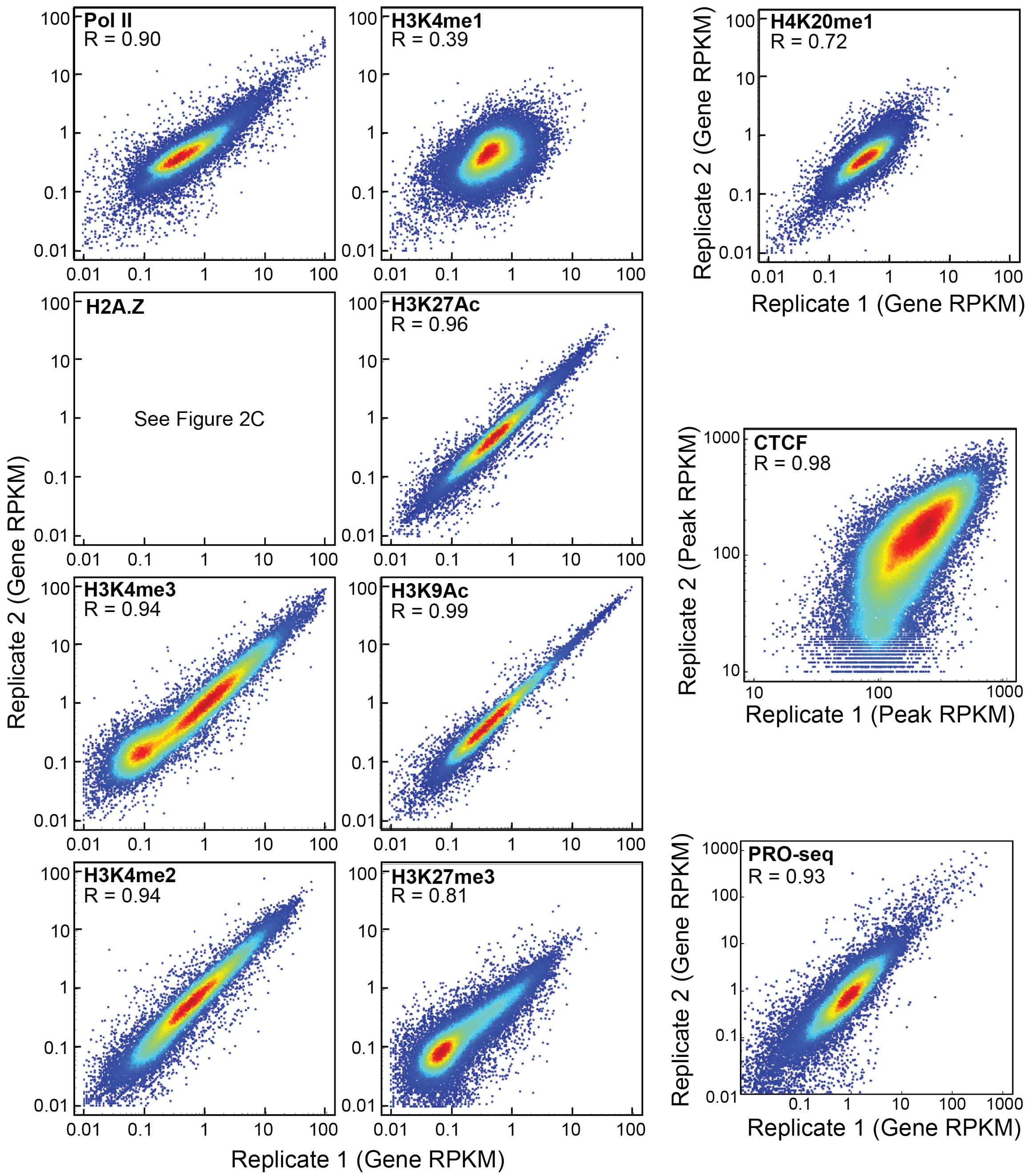

Supp. Fig. 7. Scatter plot correlation analysis for ChIP-exo biological replicates as measured by the Spearman correlation coefficient R-values (upper left corner). Related to Fig. 2c.

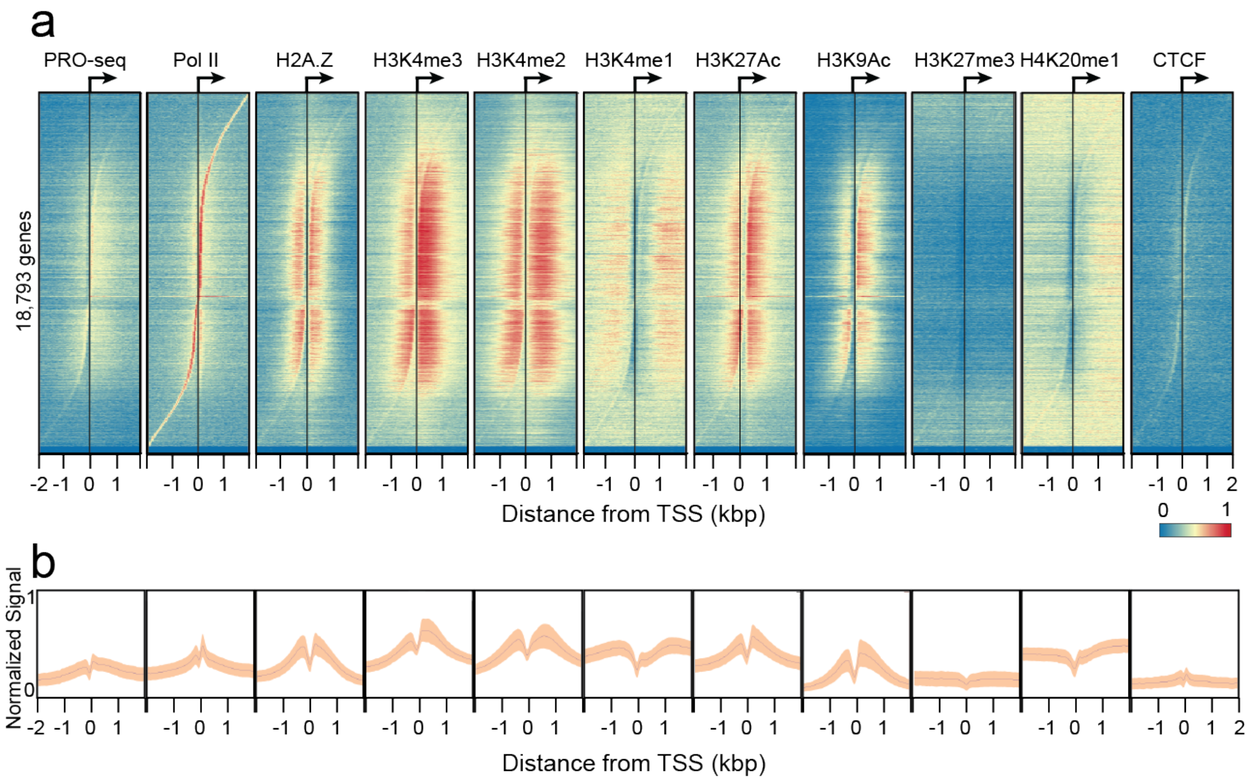

Supp. Fig. 8. ChAsE heatmap display for PRO-seq and ChIP-exo signal from merged biological replicates. Rows are linked and sorted by Pol II ChIP-exo max peak position. Related to Fig. 3a.

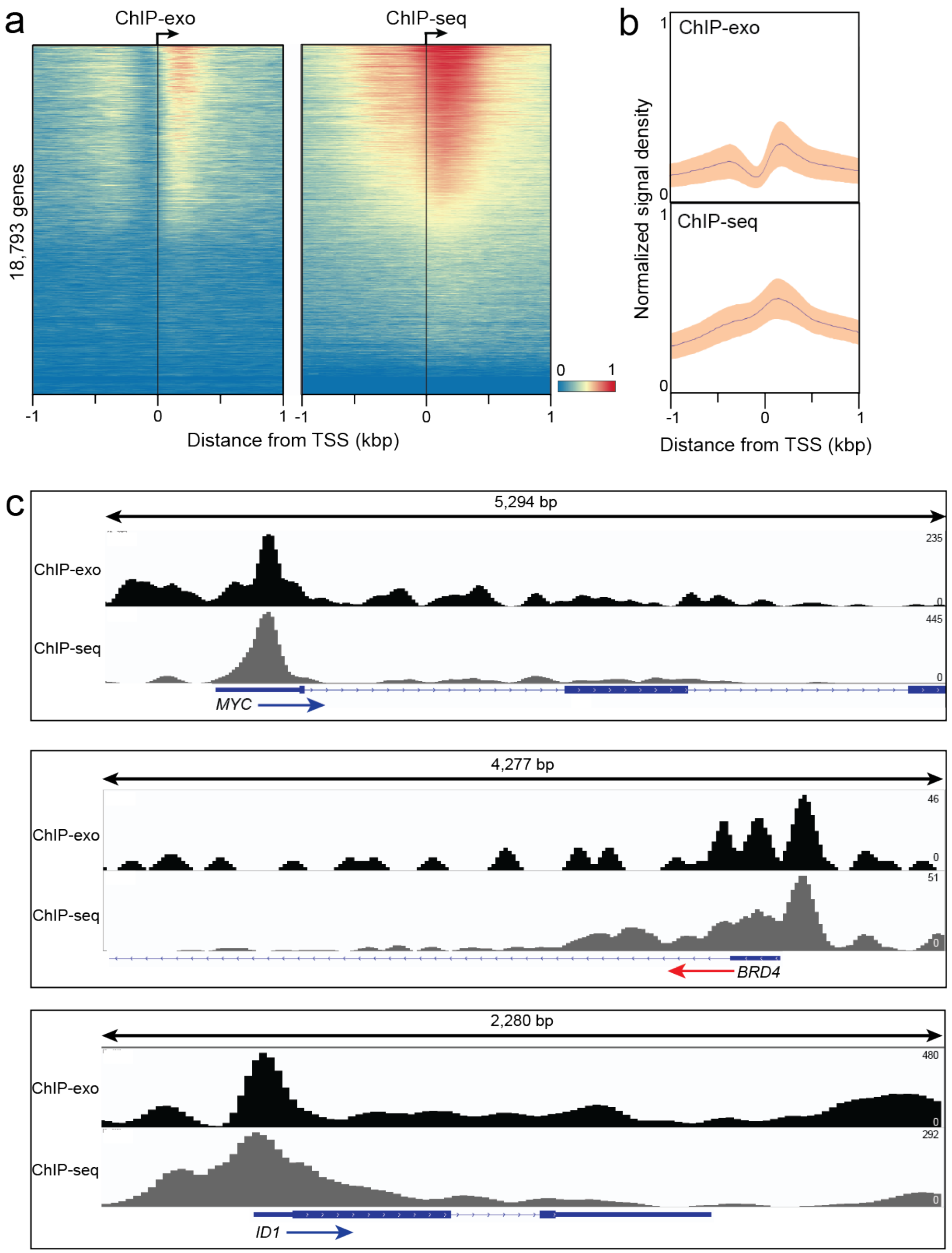

Supp. Fig. 9. Comparison of Pol II ChIP-exo and ChIP-seq resolution at promoters. Related to Fig. 3. (**a**) Row-linked heatmaps show RPKM normalized number of reads across a 2kb genomic interval in 10bp bins relative to the TSS. Heatmaps were generated from HCC1806 Pol II ChIP-exo from this study and MCF7 Pol II ChIP-seq from ENODE (encodeproject.org and SRA575091). Regions are sorted in descending order based on average row tag density. Each row represents a gene, with 18,793 genes displayed. Red and blue reflect high and low read densities, respectively. (**b**) Composite plots below each heatmap quantify the normalized tag density. The central trace denotes the average tag density for each 10bp bin and the orange fill reflects the standard deviation. (**c**) Genome browser view of Pol II ChIP-exo signal in HCC1806 cells and Pol II ChIP-seq signal in MCF7 cells for the indicated gene loci. Both data sets were processed using the same pipeline described in the methods. Their tag distributions were smoothed and RPKM normalized in the same manner using deepTOOLS.
